# Pose Mask: A Model-Based Augmentation Method for 2D Pose Estimation in Classroom Scenes Using Surveillance Images

**DOI:** 10.3390/s22218331

**Published:** 2022-10-30

**Authors:** Shichang Liu, Miao Ma, Haiyang Li, Hanyang Ning, Min Wang

**Affiliations:** 1 School of Computer Science, Shaanxi Normal University, Xi’an 710119, China; 2 Key Laboratory of Modern Teaching Technology, Ministry of Education, Xi’an 710062, China; 3 National Engineering Laboratory for Integrated Aero-Space-Ground-Ocean Big Data Application Technology, Xi’an 710072, China

**Keywords:** pose estimation, masked autoencoder, model-based augmentation, classroom scenes

## Abstract

Solid developments have been seen in deep-learning-based pose estimation, but few works have explored performance in dense crowds, such as a classroom scene; furthermore, no specific knowledge is considered in the design of image augmentation for pose estimation. A masked autoencoder was shown to have a non-negligible capability in image reconstruction, where the masking mechanism that randomly drops patches forces the model to build unknown pixels from known pixels. Inspired by this self-supervised learning method, where the restoration of the feature loss induced by the mask is consistent with tackling the occlusion problem in classroom scenarios, we discovered that the transfer performance of the pre-trained weights could be used as a model-based augmentation to overcome the intractable occlusion in classroom pose estimation. In this study, we proposed a top-down pose estimation method that utilized the natural reconstruction capability of missing information of the MAE as an effective occluded image augmentation in a pose estimation task. The difference with the original MAE was that instead of using a 75% random mask ratio, we regarded the keypoint distribution probabilistic heatmap as a reference for masking, which we named Pose Mask. To test the performance of our method in heavily occluded classroom scenes, we collected a new dataset for pose estimation in classroom scenes named Class Pose and conducted many experiments, the results of which showed promising performance.

## 1. Introduction

Student behavior analysis has been receiving significant attention as an important part of assessing student learning quality. In the booming development of computer vision, the use of human pose estimation and behavior recognition technology [1,2,3] to observe and analyze the learning behaviors and states of students has become an important issue in current educational development. As an upstream task of behavior analysis, the study of pose estimation is very important. Pose estimation is one of the fundamental tasks in computer vision and, similar to many tasks, deep-learning-based methods significantly accelerate the development progress of human pose estimation. The focus of this task is to locate the articulation points of the human body, which has a wide range of real-world applications. As a data-driven task, some early works on MPII [4] showed excellent performance, while further research based on deep convolution neural networks (CNN) achieved great progress, pushing the benchmark to practical saturation in recent years, reaching over 0.94 PCKh-0.5 [5]. Recent research and progress moved to a more complex and challenging keypoint detection dataset named MS COCO [6], which contains variations of occlusion, truncation, and scales. Many studies on the MS COCO dataset achieved state-of-the-art performance on all pose estimation benchmarks, which demonstrated the generalizability of the data. MS COCO has helped to solve the problem of pose estimation in more diverse and complex scenes to some extent, but not much work has focused on occlusion in classroom scenes as the key research object.

**Figure 1:**
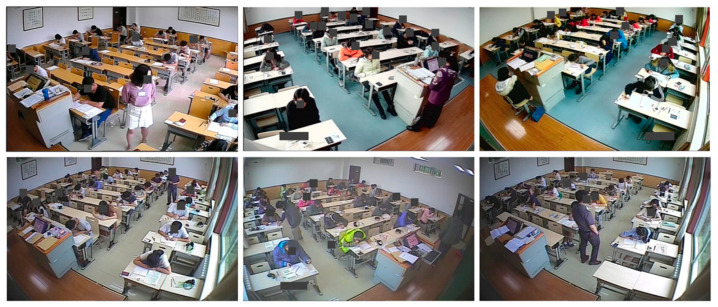
Samples in the Class Pose dataset.

In classroom scenarios, the problems of student limbs being obscured by desks and overlapping between students in classroom images can pose a considerable challenge to pose estimation, and poor accuracy of pose estimation results can also result in poor data quality for downstream tasks. Lin et al. [7] designed a behavior recognition system that used the OpenPose framework [8] to obtain skeleton data, a correction scheme was proposed to reduce false connections, and then the skeleton data was modeled into different categories and action recognition was implemented using a classifier. Xu et al. [9] proposed a method for head pose recognition based on Euler angles obtained from pose estimation. Yu et al. [10] collected a classroom scenario dataset in a video game image called SynPose and employed a generative adversarial network (GAN) [11] to generate skeletons for transfer learning. However, it is not publicly available, and thus, we cannot conduct any experiments on it. From previous studies, it can be seen that no work has fully investigated the problem of pose estimation, and the accuracy of it is non-negligible as an upstream task for subsequent action recognition. Moreover, to the best of our knowledge, there is no publicly available dataset to encourage a data-driven student pose estimation in classroom scenes. In this study, we collected surveillance image data from classrooms and proposed a new dataset named Class Pose to provide a benchmark and a method dedicated to addressing occlusion problems in classroom scenes. Since this dataset involves students’ personal information, the images have been specially processed, as shown in Figure 1.

As an important research field in computer vision, pose estimation has developed significantly. Widely used in industry in the early years, bottom-up methods such as the OpenPose framework are characterized by their real-time speed, which computes all keypoints from the whole image and groups them into individuals. As a result, the difficulty of the bottom-up method is the grouping of keypoints in the occluded and overlapping states. With the continuous improvement of computation, the top-down method that detects the human body first using an object detector and then performs single-person pose estimation is gradually becoming mainstream [12,13,14,15,16]. They can be further categorized as regression-based methods and heatmap-based methods. The regression-based methods [12,13] do not require the maintenance of high-resolution heatmaps, and thus, there is no burden in terms of computational and storage costs. However, they have an obvious disadvantage, i.e., fewer learnable features lead to poor performance in complex scenes (occlusion, motion blur, truncation, etc.). In contrast, heatmap-based methods pursue accuracy over speed; representative works include the stacked hourglass network [14], simple baselines [15], and HRNet [16]. Heatmap-based pose estimation is commonly used in scenarios with high reliance on detection results, such as providing accurate pose results for student behavior analysis, which is in line with our goal.

As classic forms of image augmentation, traditional image processing methods, such as pixel-level color space transformation and geometric transformation, show impressive performance in some computer vision tasks. These handcraft augmentations are fast and stable in most scenarios; however, in some cases, pixel-level transformation, such as channel shuffle or color jitter, which uses a single pixel as the operational unit to enrich the diversity of the training data by directly changing the pixel value, sometimes have a detrimental effect on model learning. Pixel-level transformation may break the dependency between pixels and lead to distorted features, while some geometric transformations, such as translation or Gaussian blur, can preserve the coherence between pixels and regions but can be easily learned by deep neural networks [17]. It demonstrates that these traditional image processing methods may not be sufficient to provide generalized augmentations. Model-based data augmentation compensates for the deficiencies found in traditional image processing, such as GAN, whose purpose is to expand the training set once the sample is insufficient. However, the samples generated by GAN are not controllable and the uncertainty in sample distribution will result in unstable features learned by the model and bring a huge loss of accuracy. As mentioned above, not only is no image augmentation specifically designed for the pose estimation task but the challenges in pose estimation, such as occlusion, are also not well addressed by the presence of these augmentations. In this study, we bridged this gap by proposing Pose Mask, as described in Section 2.

For image reconstruction, Kaiming et al. [18] proposed a masked autoencoder (MAE), which is an asymmetric encoder–decoder structure, to build unknown masked pixels from known pixels, which can also be seen as an extension of BERT [19]. The transfer performance in downstream tasks surpasses supervised pre-training; furthermore, an MAE can also be seen as a versatile model-based nonlinear image augmentation method [20]. Our proposed method adopts the idea that using a self-supervised MAE to pre-train on the MS COCO dataset to achieve better generalizability in the occlusion domain, which is easily controlled because both masked-out patches and occluded limb parts are consistent in terms of feature incompleteness and need to establish dependencies with the surrounding pixels. In general, the pre-trained model is used to initialize the backbone of the pose estimator and enlarge the training set.

In this study, we proposed a novel model-based image augmentation method named Pose Mask that was designed for the occlusion problem of pose estimation. The design of the Pose Mask does not exactly follow the original MAE masking strategy, which randomly masks out up to 75% of all image patches. However, we counted the distribution probabilities of the human joint points in all the data and plotted a distribution heatmap as a mask template, which served as a reference to mask only the regions where the keypoints appeared the most. Our method adopted the MS COCO dataset for pre-training the MAE that used Pose Mask. By dropping pixels on limb parts to simulate the information loss caused by occlusion and feeding the rest of the unmasked patches to the encoder, the decoder takes the output of the encoder that was spliced with the vectors, which represent the masked patches for reconstructing the occlusion part. It is worth noting that the decoder was only used in the pre-training phase and can be replaced with any architecture if transferred to downstream tasks [18]. Specifically, in pose estimation, the decoder could be replaced by a few deconvolution layers to obtain the keypoint heatmaps [15,21].

In order to prepare a reliable pathway for downstream behavior analysis, the main contributions toward solving the occlusion problem of pose estimation task in classroom scenes are summarized as follows:

1. To address the difficulty of pose estimation in classroom scenarios and to validate the effectiveness of our model-based data augmentation, we proposed a new dataset named Class Pose.
2. Inspired by the masked autoencoders in image reconstruction, we proposed a model-based data augmentation method named Pose Mask, which served to fine-tune the pose estimation model using the reconstructed images as the new training set that was generated by the MAE trained with Pose Mask.
3. A novel pose estimator was proposed, which uses ViT [22] and the early stages of ResNet [23] as the backbone, and was initialized after the weights were trained using the Pose Mask on the MS COCO dataset.

## 2. Materials and Methods

### 2.1. Overall Architecture

**Figure 2:**
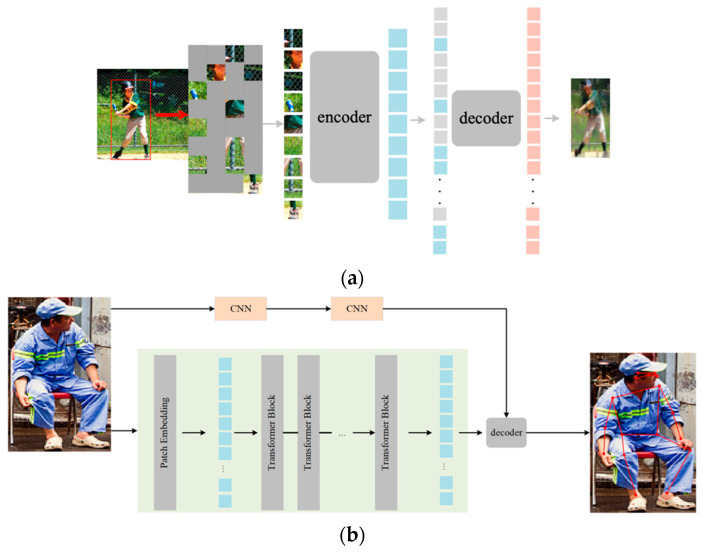
Illustration of the overall architecture of our method in classroom scenes: (**a**) pre-training pipeline of the MAE using the MS COCO train2017 dataset; (**b**) pose estimator pipeline using ViT with a SideCar CNN as a backbone and a decoder adopts deconvolution layers.

Considering the excellent transfer performance of an MAE, we pre-trained it using the MS COCO dataset (only the person category was selected), which was different from the original MAE to better adapt to the dense human pose estimation in classroom scenes, and the 75% random mask was replaced by the proposed Pose Mask. The pre-trained weights served as a model-based image augmentation for occluded scenarios and could be loaded by the ViT part of the pose estimator backbone for fine-tuning. Figure 2 illustrates the suggested architecture for 2D pose estimation based on classroom surveillance. The whole pipeline could be illustrated as pre-training of the MAE and fine-tuning of the pose estimator.

For the pre-training phase, we used cropped images from the person category from MS COCO, which contains 262,465 single-person samples, to allow the model to better fit human skeleton features, as shown in Figure 2a. First, the cropped images were masked with our proposed Pose Mask, while the remaining unmasked image patches were positionally embedded and fed into the encoder. Next, the decoder followed the same design as the MAE, and thus, the input of the decoder contained two parts: the feature vectors of the unmasked patches that were output by the encoder and the masked patches that were represented using the same learnable feature vector. Finally, a linear projection was adopted after the decoder for reconstruction, which only calculated the loss of unmasked patches.

In Figure 2b, to transfer our focus to pose estimation in classroom scenes, the same ViT backbone without the pose mask was adopted in the pose estimator design. Furthermore, we added two CNN stages to retain high-resolution features in the early stages. The ViT backbone of our pose estimator was initialized with the pre-trained weights of the MAE. The encoder of ViT was composed of several transformer blocks, which were scalable in depth. We used deconvolution layers as the decoder to generate the heatmap that fused with the reshaped SideCar feature map for each keypoint. After the proposed pose estimator was fine-tuned using the MS COCO dataset (original and reconstructed images) and 500 images from Class Pose, we tested the performance on the remaining images in Class Pose.

### 2.2. Pose Mask

The original MAE masking method involves splitting the image into patches of the same sizes first and then using positional embedding to stretch all the patches into vectors, and after shuffling patches randomly, selects the first 25% of the patches as input into the encoder, thus reducing 75% of the computational burden of the transformer blocks.

**Figure 3:**
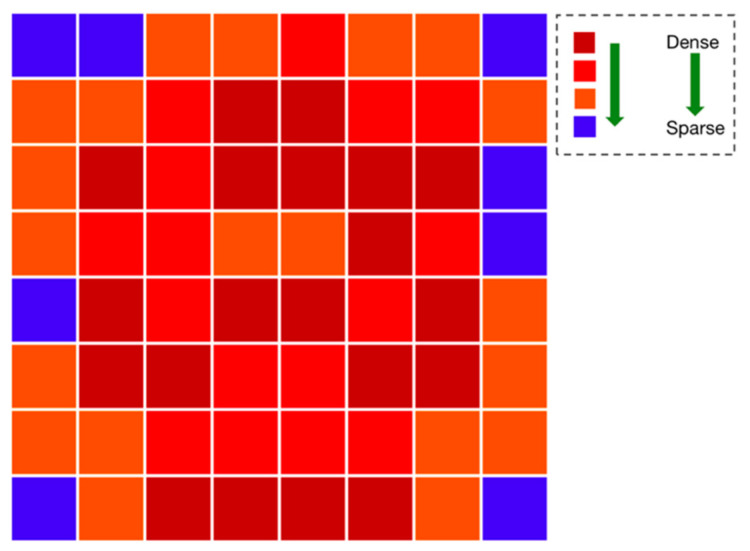
Probability heatmap of keypoint distribution.

Different from the above shuffling strategy in the original MAE, we statistically calculated the distribution of all keypoints in the MS COCO dataset, as shown in Figure 3. The distribution heatmap was formed into an 8 × 8 chessboard; from dark red to blue, the blocks indicate the decreasing distribution probability of keypoints in this region. The calculation details about the probability of each block were as follows: (1) we mapped all of the keypoints from the MS COCO dataset with different sizes to the same size, which was a 256 × 256-pixel area; (2) the 256 × 256-pixel area was further transformed to 8 × 8 blocks (32 × 32 pixels for each block), we counted the number of keypoints fallen inside each block, and sorted them in descending order; and (3) according to the number of keypoints in each block, we graded them and then divided the 64 blocks into four different probability heat levels. From high to low, the blocks appeared as follows: 20 dark red, 16 bright red, 20 orange, and 8 blue. The significance of the heat level design was that they were the reference for selecting patches in the next step of the random masking.

**Figure 4:**
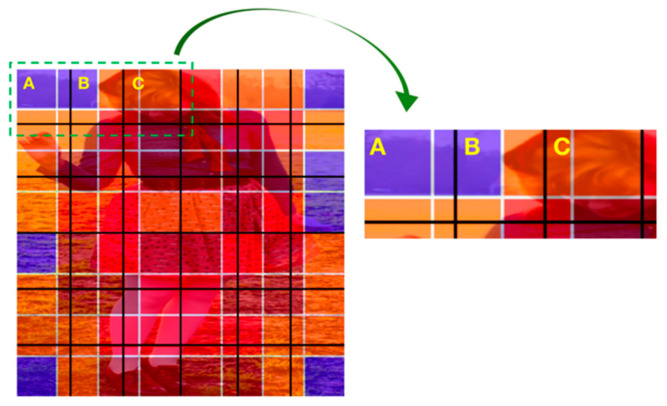
Illustration of the masking strategy.

For the masking strategy, the image was resized to a square and divided into patches before being fed into the transformer encoder; then, we mapped the probability heatmap to the same size as the input image. Therefore, the image patches overlapped the color blocks (probabilistic heatmap) with different areas. The blocks with four different colors were assigned different weights, which were set to 4, 3, 2, and 1 from high to low, respectively. Finally, a linear weighted randomization (LWR) algorithm was applied to weight the patches, which was calculated as follows:

*W_i_* = 4 × *A_i_*^DR^ + 3 × *A_i_*^BR^ + 2 × *A_i_*^O^ + 1 × *A_i_*^B^, (1)

where *W_i_* denotes the weight of the *i*th patch; *A_i_*^DR^, *A_i_*^BR^, *A_i_*^O^, and *A_i_*^B^ represent the block areas with dark red (DR), bright red (BR), orange (O), and blue (B) that the *i*th patch occupies, respectively; and the weight of the *i*th patch is the sum of areas times their weights. As shown in Figure 4, the input image was divided by several black lines, and then three patches *A*, *B*, and *C* were selected as an example, where patch *A* occupied approximately 1.3 blue blocks and 0.44 orange blocks, which had a weight of 2.18. Similarly, patch *B* had a weight of 3.33 and patch *C* had a weight of 4.2. Once the weights of all patches were obtained, we selected the patches that should be masked out, and an accumulated weight was calculated as follows:

*AW_i_* = *AW_i_*_−1_ + *W_i_*, (2)

where *AW_i_* denotes the accumulated weight of the *i*th patch position for a total of *N* patches. A random number *F* ∈ [*AW*_1_, *AW_N_*] was generated. Then, we traversed from *AW_i_* to *AW_N_* for comparison purposes; if *AW_i_* ≤ *F* < *AW_i_*_+1_, the *i*th patch was masked (when the patch was masked out, the weight range should also be removed from the random generation space). Note that the traversal operation was executed a number of times equal to 75% of the number of patches, which was the same portion of the masked patches as the original MAE. In this way, the patches with larger weights were prone to be masked, which, to some extent, gave the keypoints more chance to be masked and forced the MAE to learn the missing keypoints’ information, thus improving the generalizability of the model in heavily occluded classroom scenes. The rest of the pre-training procedure of the MAE was the same as the original.

### 2.3. The Design of Pose Estimator

The ViT part of our backbone was the same as the pre-training phase, only without masking. Many studies [24,25,26] demonstrated that early-stage CNNs perform better than a transformer, and early convolutions help transformers see better. Inspired by CBNet, an assistant backbone was used to enhance the leading backbone. Since this would result in an excessive number of parameters, for simplicity, two CNN stages were added to the side of ViT, named SideCar, which provided the feature map from the early stages of the CNN to be fused with the output heatmap from the ViT decoder. In this way, SideCar could compensate for the missing local features to some extent. The pose estimator is shown in Figure 2b. For the ViT part, an input image was divided into N × N patches and fed into the transformer blocks to extract features using self-attention operations. SideCar can be selected from a variety of CNN structures; here, we took the first two stages of ResNet-50 as the baseline.

**Figure 5:**
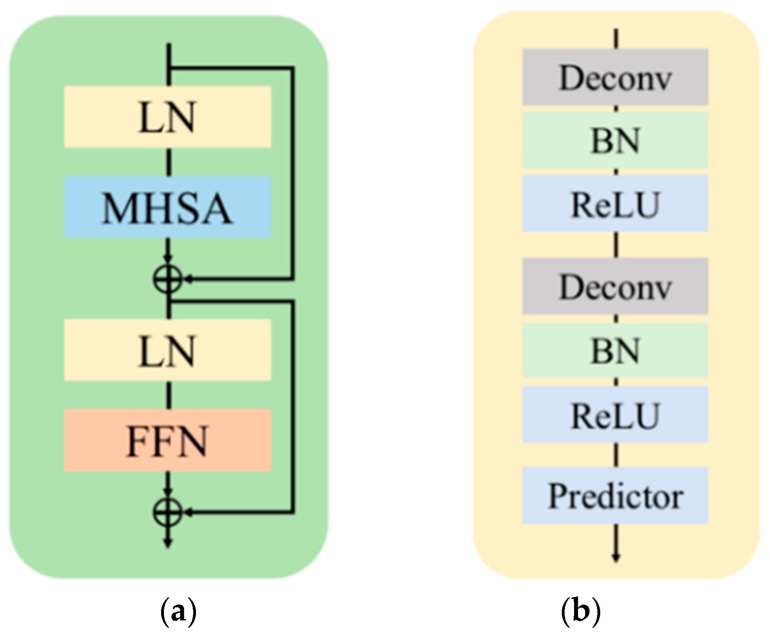
Detailed structure of the proposed pose estimator: (**a**) the structure of the transformer block, where LN, MHSA, and FFN denote the layer normalization, multi-head self-attention, and feed-forward network, respectively; (**b**) the structure of the pose estimator decoder with two deconvolution layers, where Deconv, BN, and ReLU represent the deconvolution operation, batch normalization, and ReLU activation, respectively.

The pre-trained weights of MAE were loaded to initialize the backbone of the pose estimator for fine-tuning the model on the MS COCO dataset with 500 images from Class Pose. The goal of this subsection was to provide a simple yet effective baseline that used MAE-pre-trained weights as a model-based image augmentation for pose estimation in classroom scenes. To this end, we borrowed an idea from ViTPose to implement our model, namely, we did not use any skip connections or cross-attentions; instead, several deconvolution layers and a prediction layer were appended to the backbone in order to estimate the heatmaps, as in simple baselines [15]. The design was found to be efficient and powerful, as shown in Figure 5b. Moreover, as illustrated in Figure 5a, the output of the transformer block in the backbone was calculated as follows:

*F*_0_ = PatchEmbed(*X*), (3)

*F*’*_i_*_+1_ = *F_i_* + MHSA(LN(*F_i_*)), *F_i_*_+1_ = *F*’*_i_*_+1_ + FNN(LN(*F*’*_i_*_+1_)), (4)

where *X* is the original image of a person and *F_i_* denotes the output feature of the *i*th transformer block. The output of the decoder was the heatmaps of the number of keypoints:

*H_k_* = Conv_1×1_ (Deconv(Deconv(*F_b_*))), (5)

where *H_k_* represents the heatmap of *k* channels, and *k* is the number of keypoints. *F_b_* denotes the output feature of the backbone.

### 2.4. Class Pose Dataset

#### 2.4.1. Dataset Collection

To collect the data for Class Pose, we sample 800 images from 20 real-world in-classroom surveillance videos from two perspectives. We further collected 200 more surveillance images of classrooms online. We selected Cascade R-CNN [27] with ConvNeXt-L [28] as the object detector to detect human bodies for the downstream pose estimation task. The keypoint annotations were manually labeled.

#### 2.4.2. Properties of Class Pose

Class Pose comprised 1000 images of 56,732 instances in various resolutions, making it adequate for evaluation. We summarized the properties of Class Pose into the following aspects:

**Scenes.** We collected two perspectives of 10 different classroom scenes from the common surveillance cameras in the corners of real classrooms. Each scene had 25–40 students, each sitting behind their table with serious occlusion. The 200 online images contain 10–200 persons per scene.

**Joints.** We obtained 17 joints of each instance following the MS COCO dataset 0–16, which contained the following: nose, left eye, right eye, left ear, right ear, left shoulder, right shoulder, left elbow, right elbow, left wrist, right wrist, left hip, right hip, left knee, right knee, left ankle, and right ankle.

**Annotations.** Class Pose was labeled by three people, where for each joint, the average of three labels is taken as the ground-truth. Every image came with precise annotations of visible or occluded body joints and bounding boxes.

## 3. Results

### 3.1. Implementation Details

Our model-based image augmentation method was implemented based on the official codes of the MAE [18] and ViTPose [21]. MMDetection [29] was used as the object detection toolbox of the top-down pose estimation pipeline. All the code was implemented using Python 3.8 and Pytorch 1.8. For the ablation experiments and comparisons, the input images were resized to 224 × 224 during pre-training. The AdamW optimizer was adopted with an initial learning rate of 0.003 and 0.05 as the weight decay. Using batch normalization on four RTX 3090 GPUs with CUDA 11.1, we pre-trained the MAE (ViT-Base, ViT-Large [22]) with a 75% pose-masking strategy for 2000 epochs with a batch size of 256; randomly resized cropped and horizontal flipped images were also used for pre-training image augmentation. As for fine-tuning, the pre-trained weights of the MAE were loaded to initialize the ViT part of the pose estimator backbone. Finally, we trained 210 epochs for each size of the backbone (ViT-Base, ViT-Large) with a batch size of 128, while the size of the input image was set to 256 × 192 or 384 × 288.

### 3.2. Datasets and Evaluation Criteria

The MS COCO train2017 dataset contains 118,287 images of 80 object categories in various scenes; here, we cropped all the instances of the person category using the annotation of the object detection task to provide a total of 262,465 single-person samples, which was enough for pre-training the MAE. We manually labeled 1000 Class Pose images, including 500 images for evaluation and 500 images for fine-tuning the pose estimator. We compared the AP (average precision) of the pose estimation results in various settings.

The AP was calculated as the average of precisions at different *OKS *thresholds [6], which is the area enclosed by precision (*P*), recall (*R*), and the coordinate axis at an *OKS* threshold θ:

*OKS* = Σ*_i_*[exp(−*d_i_*^2^/2*s*^2^*k_i_*^2^)δ(*v_i_* > 0)]/Σ*_i_*[δ(*v_i_* > 0)], (6)

*P* = *TP*/(*TP* + *FP*), *R* = *TP*/(*TP* + *FN*), (7)

where *OKS* represents the object keypoint similarity, *d_i_* is the Euclidean distance between each corresponding ground truth and the predicted point, and *v_i_* is the visibility sign of the ground truth. To calculate *OKS*, *d_i_* is passed through an unnormalized Gaussian with standard deviation *sk_i_*, where s denotes the object scale and *k_i_* is a per-keypoint constant that controls the decay. Formula (6) yields a keypoint similarity between 0 and 1 for each keypoint (keypoints with *v_i_* > 0 are visible keypoints). Predicted keypoints that are not visible (*v_i_* = 0) are not involved when calculating *OKS*. *OKS* tends to 1 when the predicted result is closer to the ground truth and 0 vice versa. The way that precision and recall are calculated use *OKS* = θ as the threshold, that is, *TP* represents a positive predicted result with an *OKS* ≥ θ, and similarly for other cases.

### 3.3. Ablation Study

#### 3.3.1. Effect of Pre-Training the MAE via Pose Mask

To compare Pose Mask with the original masking strategy, we also pre-trained the MAE with MS COCO using the default settings and added reconstructed images to fine-tune the proposed model. The results for the Class Pose validation set are listed in Table 1.

**Figure 6:**
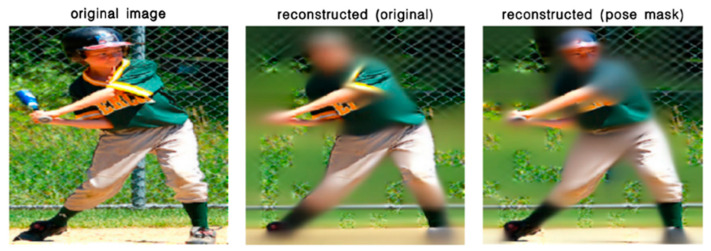
Reconstructed images with different masking strategies.

As seen in Table 1, both masking strategies effectively improved the performance of the model compared with the ViT backbone without pre-training using the MAE. The use of Pose Mask produced an obvious improvement, which indicated the weighted random masking strategy using a probabilistic heatmap caused the model to learn features of the masked-out keypoints. Hence, the masking strategy could accurately simulate the occlusion problem in classroom scenarios. The reconstructed images of two masking strategies are visualized in Figure 6, where different masking strategies did not affect the performance of image reconstruction since the main aim of the MAE is to restore the missing image information. However, Pose Mask made the model focus on the hot areas where the limbs may be occluded, which had a positive influence when transferring to the pose estimation task.

#### 3.3.2. Effect of SideCar

In terms of the fundamentals of a CNN, the inductive bias induced by convolutions, namely, translation invariance and locality, were found to be effective for visual data over many years of research. The advantage of a CNN regarding local features can be added as compensation to the heatmap of the ViT part’s output. There are various choices available regarding the SideCar structure, and here we chose three common CNN structures for the associated experiments. The results are listed in Table 2.

As seen in Table 2, SideCar performed the best as a useful feature compensator when the number of stages was two. This was because the semantic features of later stages were similar to the ViT part’s output, which did not compensate well, while the features in the earliest stage could not eliminate the interference from the background, and thus, had a negative impact. After replacing the CNN architecture with each of several stronger backbones, namely, ResNet-152, ResNeSt-50, and Res2Net-50, significant improvements were also obtained. The experimental results showed the effectiveness of SideCar. Note that the 101-layer structure had the same stage 1 and stage 2 as the 50-layer structure; therefore, we did not test it.

#### 3.3.3. Comparisons with Mainstream Pose Estimators

To verify the superiority of our pose estimation approach in heavily occluded classroom scenes, we retrained some mainstream models using the same training set (MS COCO train2017 and 500 images from Class Pose) and validated it using Class Pose. The corresponding experimental results are listed in Table 3, where the proposed method surpassed the mainstream pose estimator.

#### 3.3.4. Comparisons with Potential Image Augmentation Methods

The improvement achieved when using the Pose Mask demonstrated its excellent prediction ability for the missing keypoints, but as a form of model-based image augmentation, its effectiveness on the image itself is more noteworthy. Therefore, we use reconstructed images from MS COCO using the original MAE to compare with our method, and the results can be seen in Table 4. Moreover, we compared it with CycleGAN [34], which is also a model-based form of augmentation. Other methods based on traditional image processing verified the impacts on model performance.

After comparing Pose Mask with many traditional image augmentation methods and previous model-based approaches, it is easy to see that our model truly enhanced the pose images with an observable improvement. Note that the tested model was ViTPose-Large + ResNet50 with two stages.

### 3.4. Visualization

**Figure 7:**
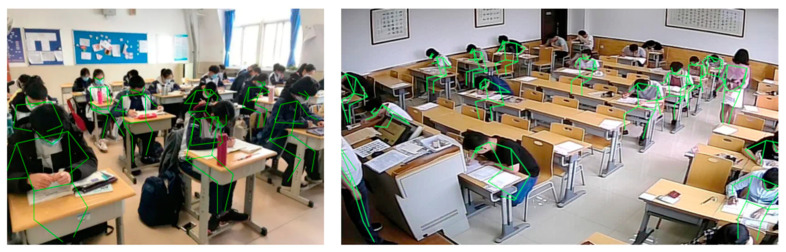
Pose estimation results of our method (detected bounding boxes with low scores were removed).

In order to visually test the effectiveness of our method, we visualized the pose estimation results for some in-classroom images. In Figure 7, it is clear that even the lower half of the body was also well detected in areas that were heavily obscured, such as sitting behind a desk.

## 4. Discussion

### 4.1. Issues with Pre-Trained Data

In the original MAE, the training dataset used for image reconstruction was ImageNet-1K (IN-1K), which contains about 1.2 million training images, and thus, a sufficient amount of data ensured the generalization of pre-training. The MS COCO dataset contains 118,287 training images, which have already been used for object detection, pose estimation, image segmentation, etc. We cropped person instances using object detection annotations to obtain a total of 262,465 images. After the MAE was trained for 2000 epochs, its weights were used to generate reconstructed images of the same amount of data from MS COCO. Compared with IN-1K, only 22% of the data was used to pre-train the MAE using Pose Mask, which raised the concern of insufficient data. As shown in Table 1, regardless of the masking strategy, we observed that the pre-training of MAE for human images was stronger than that of IN-1K pre-training, even with nearly 80% less data. We think this was because the single class of images made the model specialized at recovering occluded limbs. The impact of data quantity for pre-training is worth further exploring; therefore, we will test the performance changes by adding more human images in future work.

### 4.2. Consistency of the Proposed Method

As a model-based form of image augmentation, the Pose Mask pipeline is not end-to-end, unlike the traditional image augmentation methods that can transform images online during training. The method we proposed serves as pre-trained weights for initializing the pose estimator backbone and provides extra data to enlarge training samples. However, such an approach limits the scope of the augment with fixed weights, and data preferences work only for the downstream pose estimation task. Since ViT exists in both the MAE and the pose estimator, we considered changing this data-expanding image augmentation method to an online mode by setting a switch to control the task to be performed. The MAE has the same structure as our pose estimator, which obtains the reconstructed images when a mini-batch of images is first input. The switch is adjusted to feed the original images and the reconstructed images into the backbone with SideCar for fine-tuning. This implementation is feasible, but it involves generating reconstructed images with fixed pre-trained weights, and fine-tuning is required to produce updated weights. Although their backbone structures are similar, they cannot share the same parameters, which produces a double burden on video memory and processing speed.

### 4.3. Contribution to Downstream Tasks

In downstream tasks, specifically, in behavior analysis or action recognition, the coordinates of the keypoints provided as input by pose estimation can affect the final performance. For instance, by using several sets of keypoint coordinates, we can calculate the angle of the relevant body part and further predict the pose of the person. In this case, the precision of joints affects the accuracy of the calculation of the angle, and thus, the judgment of the action. As illustrated in Figure 7, with the help of Class Pose, the pose estimator can predict large areas of occluded body parts, and thus, serves well for downstream tasks.

## 5. Conclusions

In this paper, we first present a novel masking strategy of an MAE for pose estimation in classroom occluded scenes named Pose Mask. The use of a probabilistic heatmap makes the MAE focus on learning the missing information of the skeleton, thus providing a better representation of the occluded feature. We also improved the performance of ViT by adding SideCar as a feature compensator. To test the effectiveness of our proposed method, we collected a pose dataset from real-world surveillance cameras in classrooms. Our experimental results showed promising performance on the Class Pose dataset. In future work, we will continue to expand the Class Pose dataset. Furthermore, we think there is much room for improvement in the design of SideCar, and an end-to-end data augmentation method based on Pose Mask is also a key research direction.

## Figures and Tables

**Table 1:** Influences of pre-trained weights using different masking strategies on pose estimation.

Pre-Training Strategy	Backbone Choice [22]	AP on Class Pose (%)
MAE-Original *	ViT-B	59.3
MAE-Pose Mask *	ViT-B	60.2
MAE-Original *	ViT-L	60.5
MAE-Pose Mask *	ViT-L	61.8
MAE IN-1K ^1^	ViT-B	59.1
MAE IN-1K	ViT-L	59.9
NO MAE ^2^	ViT-B	57.3
NO MAE	ViT-L	58.8

The AP results were obtained using the fine-tuned weights with MS COCO and part of Class Pose. * denotes the training data were person instances in MS COCO; ^1^ IN-1K stands for the MAE pre-trained with ImageNet-1K; ^2^ NO MAE represents the pose estimator without pre-trained weights in the MAE in the fine-tuning stage.

**Table 2:** Different selections of SideCar structures.

Model	Stages	AP on Class Pose (%)
ResNet-50	1	61.6
ResNet-50	2	62.0
ResNet-50	3	62.1
ResNet-50	4	61.9
ResNet-152	2	62.7
ResNeSt-50 [30]	2	62.5
Res2Net-50 [31]	2	62.4

**Table 3:** Comparisons with mainstream models.

Model	Input Size	AP on Class Pose (%)
Simple Baselines (ResNet-152)	256 × 192	52.3
Simple Baselines (ResNet-152)	384 × 288	54.6
HRNet-W48 [16]	256 × 192	54.4
HRNet-W48	384 × 288	55.8
HRNet W48 + DarkPose [32]	384 × 288	57.8
UDP-Pose-PSA [33]	256 × 192	58.7
UDP-Pose-PSA	384 × 288	59.4
ViTPose	256 × 192	60.1
ViTPose	384 × 288	60.5
ViTPose + PM ^1^	384 × 288	61.8
ViTPose + SideCar ^2^	384 × 288	60.9
ViTPose + SideCar + PM	384 × 288	62.1

^1^ denotes loading pre-trained weights using Pose Mask. ^2^ stands for the base model with stages 1 and 2 of ResNet-50. All the above models had extra fine-tuning data that was reconstructed using the MAE with Pose Mask.

**Table 4:** Comparisons with other image augmentations.

Augmentation	Type	AP on Class Pose (%)
N/A	N/A	59.1
MAE with reconstructed images ^1^	Model-based	61.3
MAE with reconstructed images (PM) ^2^	Model-based	62.1
CycleGAN	Model-based	59.3
Horizontal flip	Traditional	59.7
Vertical flip	Traditional	57.9
Random rotate	Traditional	58.4
Random crop	Traditional	59.2
Color jitter	Traditional	59.3
Channel shuffle	Traditional	58.8
Gaussian blur	Traditional	58.5
Cutout	Traditional	59.4

^1^ denotes loading pre-trained weights from the original MAE. ^2^ denotes loading pre-trained weights from PM.

## Data Availability

People-centric datasets pose ethical challenges. The dataset presented in this study will be released after full desensitization.
